# Association between gingivitis, tooth loss and cardiovascular risk: Insights from a 10-year nationwide cohort study of 3.7 million Koreans

**DOI:** 10.1371/journal.pone.0308250

**Published:** 2024-08-02

**Authors:** Seung Yeon Lee

**Affiliations:** 1 Seoul National University Hospital, Seoul, Republic of Korea; 2 Seoul Metropolitan Government-Seoul National University Boramae Medical Center, Seoul, Republic of Korea; Universidad Privada San Juan Bautista, PERU

## Abstract

**Background:**

While studies have suggested an association between periodontal disease and an increased risk of cardiovascular disease, the strength of this association and its specific links to various types of cardiovascular disease have not been thoroughly investigated. This study aimed to examine how gingivitis and tooth loss affect cardiovascular diseases, probing their individual impacts.

**Methods:**

A retrospective cohort study was conducted, encompassing 3,779,490 individuals with no history of cardiovascular disease, utilizing data from the National Health Examination and the Korean National Health Insurance database from 2006 to 2019. Cox proportional hazards models were applied to estimate the association between tooth loss, gingivitis, and cardiovascular disease.

**Results:**

Following a median follow-up of 10.38 years, 17,942 new cardiovascular disease cases were identified, comprising 10,224 cases of angina pectoris, 6,182 cases of acute myocardial infarction, and 9,536 cases of stroke. It was observed that the risk of stroke was significantly higher in the tooth loss group compared to the control group (adjusted hazard ratio [aHR]: 1.09, 95% confidence interval [CI]: 1.04–1.15). In the group with gingivitis and tooth loss, the risk of stroke and cardiovascular disease was significantly higher than in the control group (aHR: 1.12, 95% CI: 1.04–1.20; aHR: 1.08, 95% CI: 1.03–1.14). The gingivitis group exhibited a higher risk associated with stroke (aHR: 1.05, 95% CI: 1.01–1.10) among individuals aged 50 and above. However, statistically significant associations between periodontal disease and angina pectoris were not observed, nor between periodontal disease and acute myocardial infarction except among those aged above 50. Furthermore, the association between periodontal disease and cardiovascular disease was found to be stronger among individuals over the age of 50, males, those with obesity, and smokers compared to the control group.

**Conclusions:**

Our results emphasize the association of tooth loss and gingivitis with cardiovascular disease, specifically stroke, underlining the critical need for preventive oral healthcare. Tailored interventions are necessary to reduce the heightened risk of cardiovascular disease events, especially stroke, among older, obese individuals and smokers.

## Introduction

Periodontal diseases (PD), including dental caries, periodontitis, and tooth loss, affect more than 44.5% of the global population according to the 2019 Global Burden of Disease [1]. Approximately 42% of adults in the United States suffered from periodontitis and 11% suffered from tooth loss from 2009 to 2014 [2]. In South Korea, PD is ranked as the most frequent outpatient disease, with an estimated 17.4 million cases in 2021 [3]. PD is an inflammatory disease that progresses to gingivitis, periodontitis, and tooth loss, and the progression of this disease is caused by the activity of periodontal pathogens such as *Porphyromonas gingivalis* (*P*. *gingivalis*), *Aggregatibacter actinomycetemcomitans* (*A*. *a*), *Tannerella forsythia* (*T*. *Forsythia*) and *Treponema denticola* (*T*. *denticola*) [4]. Although most cases of PD are mild and preventable [5], they can be associated with chronic diseases such as cardiovascular disease (CVD) [6, 7].

In recent decade, studies on link between PD and CVD have examined worldwide. Previous case-control studies [8, 9], prospective cohort studies [6, 10–12], and retrospective cohort studies [13–17] reported that PD is associated with a high risk of CVD and Coronary Heart Disease (CHD). Studies have reported that oral bacteria such as *P*. *gingivalis* act on the immune system to coexist with the microbiome and cause inflammatory diseases [18–21], and this chronic immune response can affect the whole body. Another explanation for the observed association could be that the two disease entities share common risk factors such as smoking, diabetes, age, low socioeconomic status, physical inactivity, dyslipidemias, and alcohol consumption [22].

CVD continues to be the primary contributor to global disease burden. Its prevalence continues to increase over several decades in nearly all countries except those in the high-income category [23, 24]. In South Korea, CVD had been the second common cause of death in 2021, reporting 61.5 mortality (per 100,000 population) based on data on causes of death from the National Statistical Office.

Although growing evidence suggests a link between PD and CVD, but the strength of the association and the specific types of CVD affected with adjustment by multiple confounders are still unclear. Previous studies investigating PD and CVD risk had several limitations, such as lack of information on multiple confounding factors [8, 9, 11, 12, 15], use of self-reported diagnosis of PD [6, 9, 13], insufficient number of cases [8, 9, 13, 15, 17], and population of specific age groups [6, 9, 13, 17]. In contrast to previous studies that investigated the impact of periodontitis [6, 9, 12, 14, 15] and tooth loss [6, 11, 25] as primary explanatory variables on CVD, this study defined gingivitis and tooth loss as the oral health variables and explored the effects of both conditions. Unlike periodontitis, which represents the progression stages of PD, defining gingivitis expands the scope of investigation, enabling the capture of the entire spectrum of periodontal pathology, including early-stage symptoms. Therefore, gingivitis may play a more sensitive role in assessing oral health-related risk factors associated with CVD. Moreover, studies analyzing the risk of CVD through tooth loss are limited by their restriction to specific genders [6] and lack comprehensive investigation of CVD [11, 25].

As Korean government examines a mandatory health examination including oral screening for all individuals above 20 years old, standardized data of all examinees, totaling 3.7 million, were collected from dentists, consisting of two PD—gingivitis and tooth loss. By combining the National Health Insurance claim data of these examinees, the association between PD and total CVD, angina pectoris (AP), acute myocardial infarction (AMI), and stroke (ST) risks was investigated on overall and sub groups. This study aimed to investigate the impact of gingivitis and tooth loss on CVD, as well as their specific effects on each CVD. Identifying individuals at higher risk through early onset and progression of PD could facilitate early intervention and promote early prevention strategies to mitigate CVD risks.

## Methods

### 1. Study design and participants

This study was designed a population-based, retrospective cohort study using National Health Insurance Service (NHIS) database from 2006 to 2019 in Korea. The NHIS serves as the predominant insurance provider in Korea covering almost all citizens and playing a role in ensuring the accessibility of healthcare services to the population. Individuals enrolled in the insurance system are eligible to receive standardized medical examinations for health screening every two years. These examinations involve measurements of height, weight, and blood pressure (BP), fasting blood sugar (FBS), and also include oral examination.

In South Korea, since 1980, the National Health Examination Program for all citizens has been implemented under the National Health Screening Act, aimed at early detection and treatment of diseases. As a result, uniquely worldwide, a national health screening system spanning the entire lifespan, divided into infants, students, and adults, has been introduced, with the oral examination system also established as part of it, aimed at early detection and treatment of oral diseases [26]. The examination institution refers to designated dental clinics where at least one dentist who has completed the oral examination institution education program designated by the Minister of Health and Welfare, along with at least one nurse or dental hygienist, works, as well as hospitals or public health centers with dental departments established, or general examination institutions that employ dentists solely for the purpose of oral examinations. The procedure involves completing a personal medical history questionnaire, undergoing an oral examination by a dentist, and then receiving individualized examination results notification and counseling.

**Fig 1:**
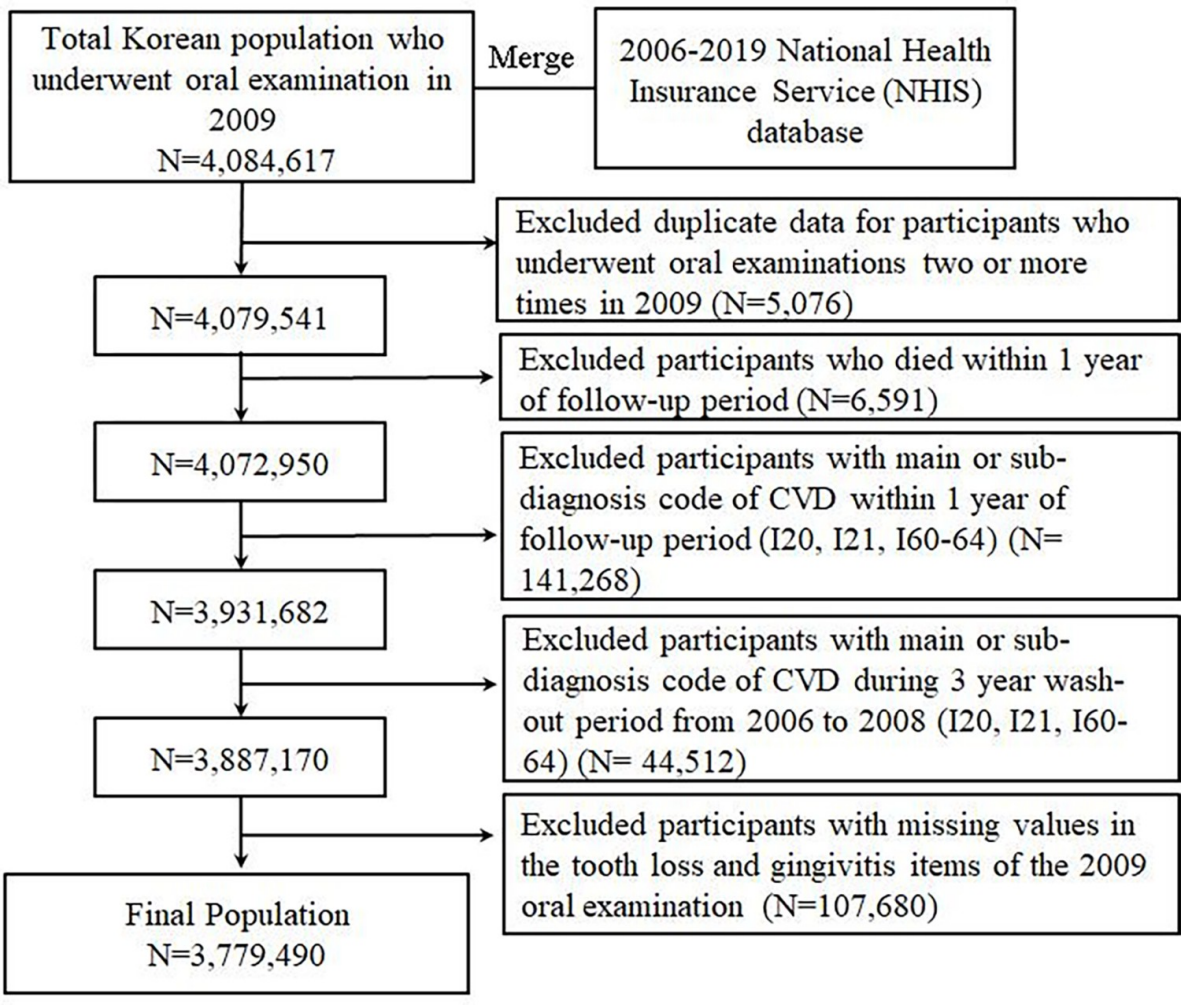
Flowchart of the study population.

**Fig 2:**
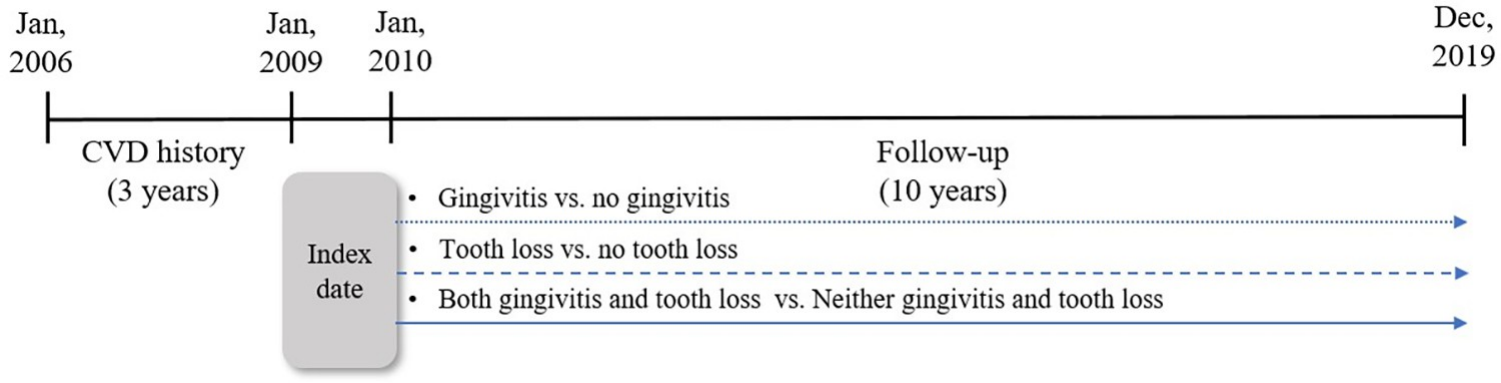
Cohort study period.

The retrospective cohort consisted of 3,779,490 adults aged 20 years or older who examined oral screening between January 1, 2009 and December 31, 2009. Based on the entire Korean population who received oral examinations in 2009, claims data including medical records from the NHIS and health examination data from 2006 to 2019 were integrated. The index date was the date of the first oral examination in 2009, followed up for 10 years. During the year 2009, individuals who underwent oral examinations two or more times within a year were identified, and consequently, 5,076 of duplicated data beyond the initial instance were excluded Moreover, participants who died within 1 year (n = 6,591) and those diagnosed with CVD as a primary or secondary disease according to ICD-10 codes within 1 year after follow-up (n = 141,268) were excluded. The medical utilization history for the previous three years, retrievable through the cohort constructed based on the registration date of the study, was set as the washout period for medical history assessment. During the washout period of the study subjects from 2006 to 2008, patients (n = 44,512) with CVD diagnosis codes identified at least once as primary or secondary diseases were considered CVD patients and thus excluded from the study subjects. Finally, 107,680 participants with missing values in the independent variables, tooth loss, and gingivitis items in the oral examination data, were excluded (Figs 1 and 2). This study was approved by the Institutional Review Board at Korea University (KUIRB-2021-0131-02). The methods were performed in accordance with relevant regulations. Informed consent was waived because the NHIS database contains publicly available anonymized data.

### 2. Assessment of PD and CVD

Periodontal disease refers to a series of inflammatory conditions affecting the tissues surrounding and supporting the teeth, including the gums, periodontal ligament, and alveolar bone. This disease typically begins with gingivitis, an inflammation of the gums caused by plaque accumulation, and can progress to more severe forms such as periodontitis. Periodontitis involves inflammation extending deep into the supporting structures of the teeth, resulting in bone loss and potential tooth loss [2, 4, 5]. In the ICD-10, periodontal disease is classified under code K05. Specifically, it is categorized into gingivitis (K05.0), periodontal disease (K05.1), aggressive periodontitis (K05.2), chronic periodontitis (K05.3), periodontosis (K05.4), other periodontal diseases (K05.5), and unspecified periodontal disease (K05.6).

Since most dental treatments are included in the non-coverage category, there is a problem that most oral diseases using health insurance claim data are omitted as treatment codes, so we used oral examination data which is conducted by dentists once every two years to detect oral diseases such as gingivitis and tooth loss at an early stage for adults in Korea. During the examination, gingivitis and tooth loss were diagnosed using probe and teethridge by a dentist. Adults who underwent a nationwide oral examination from January to December 2009 were determined to have each periodontal disease according to the criteria below. All independent variables were binary. Additionally, the results were derived by processing the group into a group with both gingivitis and tooth loss and a group with neither.

#### Gingivitis

Gingivitis was determined by visual inspection of gingival bleeding or gingival enlargement in the following cases: (1) Cases where the gums show slight redness and bleeding upon insertion of the probe; (2) Cases where the gums show overall severe inflammation, color change, loss of stippling can be observed, and spontaneous bleeding occurs. If there were no external signs of inflammation or bleeding, it was determined that there was no gingivitis.

#### Tooth loss

Tooth loss was determined in the following cases: (1) When one or more teeth are lost due to dental caries; (2) When restoration of function is necessary through tooth restoration. If there was an implant or processed tooth that did not require additional treatment, it was not judged as tooth loss.

#### CVD

The outcomes were defined as new CVD cases during a 10-year follow-up period. CVD cases was defined as CVD diagnosis (I20, I21, I60-64) according to the International Classification of Diseases (ICD)-10 between January 1, 2010, and December 31, 2019. Each disease, AP (I20), AMI (I21), and ST (I60-64), was further analyzed.

### 3. Potential confounders

Detailed data on demographic factors (age, sex, income level), clinical factors and comorbidities [body mass index (BMI), BP, FBS, total cholesterol (TC)], and health behaviors (smoking history, alcohol intake per week, physical activity per week) were adjusted. The BMI was categorized as underweight, normal, overweight, and obese (<18.5, 18.5–22.9, 23.0–24.9, ≧25) [27]. The BP was categorized as normal and hypertension (SBP<139 and DBP<89, 140≤SBP or 90≤DBP) [28]. The FBS was categorized as normal, pre-diabetes, and diabetes (<100, 100–125, ≧126) [29]. The TC was categorized as normal and dyslipidemia (<240, ≧240) [30]. The smoking history was categorized as never smoker, former smoker, and current smoker. The alcohol intake classified as 0, 1–2, and ≧3 times per a week. The physical activity was categorized as 0, 1–2, 3–4, and ≧5 times per a week.

### 4. Statistical analysis

The student t-test for continuous variables and Chi-square test for categorical variables were performed to compare the general characteristics of participants with PD and without disease. Continuous variables were presented as means ± standard deviation (STD) and categorical variables were presented as number (%). Multivariable Cox proportional hazard regression model was performed to calculate adjusted hazard ratios (aHR) and 95% confidence intervals (CI) of PD and CVD under different periodontal status. The model was adjusted for ten covariates (age, sex, income level, BMI, hypertension, diabetes, dyslipidemia, smoking history, alcohol intake and physical activity). All statistical analyses were performed using SAS Enterprise Guide version 7.15 (SAS Institute, Inc., Cary, NC, USA). Statistical significance was set at a two-sided p-value < 0.05.

## Results

### 1. General characteristics

At the baseline, of 3,779,490 participants, 984,192 had gingivitis (26.0%), 683,630 had tooth loss (18.1%), and 245,675 had both (6.5%). Participants with tooth loss at baseline were older (62.6±13.1), male, lowest income level (16.3%) and had higher BP, BMI, FBS and TC than participant without tooth loss. Additionally, participants with tooth loss more likely to be current smokers (33.4%), had higher frequency of alcohol (≧3 per a week, 16.2%) than participant without tooth loss. The gingivitis group comparing to non-gingivitis showed similar trends to the tooth loss group. Baseline characteristics of the participants are summarized in Table 1.

**Table 1:** Characteristics of study population (n = 3,779,490).

		Gingivitis		Tooth loss		Gingivitis and Tooth loss	
		No (n = 2,795,298)	Yes (n = 984,192)	No (n = 3,095,860)	Yes (n = 683,630)	Neither (n = 2,357,343)	Both (n = 245,675)
Age							
	Mean±STD (year)	56.0±12.8	58.5±12.8	55.4±12.4	62.6±13.1	54.9±12.5	64.0±12.9
Sex							
	Male	1,698,952 (60.8)	620,714 (60.8)	1,888,839 (61.0)	430,827 (63.0)	1,424,681 (60.4)	156,556 (63.7)
	Female	1,096,346 (39.2)	363,478 (39.2)	1,207,021 (39.0)	252,803 (37.0)	932,662 (39.6)	89,119 (36.3)
Income level							
	Fifth quintile (high)	709,855 (26.4)	218,919 (23.2)	780,542 (26.3)	148,232 (22.4)	611,163 (27.0)	49,540 (20.8)
	Fourth quintile	683,350 (25.4)	229,911 (24.3)	764,452 (25.7)	148,809 (22.4)	587,522 (26.0)	52,981 (22.2)
	Third quintile	558,185 (20.8)	201,303 (21.3)	621,481 (20.9)	138,007 (20.8)	470,541 (20.8)	50,363 (21.1)
	Second quintile	419,603 (15.6)	161,191 (17.1)	460,765 (15.5)	120,029 (18.1)	343,450 (15.2)	43,876 (18.4)
	First quintile (low)	317,585 (11.8)	134,335 (14.2)	343,928 (11.6)	107,992 (16.3)	251,083 (11.1)	41,490 (17.4)
BMI (kg/㎡)							
	Mean±STD	23.5±3.2	23.8±3.2	23.5±3.2	23.9±3.2	23.4±3.2	24.0±3.2
	<25 (Normal)	1,930,426 (69.8)	653,126 (66.9)	2,138,792 (69.8)	444,760 (65.5)	1,643,623 (70.5)	157,957 (64.7)
	≧25 (Obese)	836,311 (30.2)	323,029 (33.1)	924,999 (30.2)	234,341 (34.5)	688,118 (29.5)	86,148 (35.3)
Alcohol intake/week							
	0	1,183,791 (43.7)	420,318 (43.6)	1,293,907 (43.0)	310,202 (46.5)	986,049 (43.1)	112,460 (46.8)
	1–2	1,190,596 (43.9)	398,874 (41.4)	1,340,164 (44.6)	249,306 (37.4)	1,027,224 (45.0)	85,934 (35.7)
	≧3	337,864 (12.5)	144,201 (15.0)	374,308 (12.4)	107,757 (16.2)	272,177 (11.9)	42,070 (17.5)
Smoking							
	Never smoker	1,511,223 (55.1)	499,857 (51.4)	1,673,011 (55.0)	338,069 (50.1)	1,292,169 (55.9)	119,015 (49.0)
	Former smoker	446,276 (16.3)	152,483 (15.7)	486,825 (16.0)	111,934 (16.6)	373,614 (16.2)	39,272 (16.2)
	Current smoker	787,629 (28.7)	319,486 (32.9)	881,936 (29.0)	225,179 (33.4)	647,001 (28.0)	84,551 (34.8)
Physical activity/week							
	0	1,165,813 (43.6)	433,519 (45.4)	1,285,311 (43.3)	314,021 (47.7)	968,828 (43.0)	117,036 (49.2)
	1–2	621,034 (23.2)	209,295 (21.9)	699,754 (23.6)	130,575 (19.8)	535,701 (23.8)	45,242 (19.0)
	3–4	408,010 (15.3)	139,871 (14.6)	456,754 (15.4)	91,127 (13.8)	348,580 (15.5)	31,697 (13.3)
	≧5	480,285 (18.0)	172,764 (18.1)	529,966 (17.8)	123,083 (18.7)	401,341 (17.8)	44,139 (18.5)
Blood pressure (mmHg)							
	SBP, Mean±STD	120.6±14.0	121.9±14.4	120.4±13.9	123.7±14.9	120.1±13.8	124.2±15.2
	DBP, Mean±STD	75.7±9.6	76.3±9.8	75.5±9.5	77.2±9.9	75.4±9.5	77.4±10.0
	SBP<139 and DBP<89 (Normal)	2,445,999 (88.4)	841,748 (86.1)	2,722,724 (88.8)	565,023 (83.2)	2,081,175 (89.3)	200,199 (82.0)
	140≤SBP or 90≤DBP (Hypertension)	320,963 (11.6)	135,484 (13.9)	342,211 (11.2)	114,236 (16.8)	250,792 (10.8)	44,065 (18.0)
FBS (mg/dL)							
	Mean±STD	94.8±20.5	96.4±23.1	94.3±19.9	99.1±26.0	94.1±19.5	99.9±27.3
	<126 (Normal)	2,648,252 (95.7)	924,000 (94.6)	2,943,918 (96.1)	628,334 (92.5)	2,244,237 (96.2)	224,319 (91.8)
	≧126 (Diabetes)	118,598 (4.3)	53,215 (5.5)	120,908 (4.0)	50,905 (7.5)	87,624 (3.8)	19,931 (8.2)
Total cholesterol (mg/dL)							
	Mean±STD	193.2±36.9	195.5±39.4	193.2±36.9	196.4±40.8	192.7±36.5	197.5±43.5
	<240 (Normal)	2,498,347 (90.3)	870,276 (89.1)	2,767,977 (90.3)	600,646 (88.4)	2,112,073 (90.6)	214,372 (87.8)
	≧240 (Dyslipidemia)	268,567 (9.7)	106,928 (10.9)	296,903 (9.7)	78,592 (11.6)	219,853 (9.4)	29,878 (12.2)

Values are presented as number (%) unless otherwise specified; STD, standard deviation; SBP/DBP, systolic blood pressure/diastolic blood pressure; BMI, body mass index; FBS, fasting blood sugar

### 2. Risks (aHR) of CVD in patients with gingivitis and tooth loss

After a median of 10.38 years, 17,942 new CVD cases, 10,224 AP cases, 6,182 AMI cases, and 9,536 ST cases were identified. The risk of ST was significantly higher in the tooth loss group than the control group after adjustment (aHR: 1.09, 95% CI: 1.04–1.15). In the group with both gingivitis and tooth loss (G&T), the risk of ST and CVD were higher than the control group significantly (aHR: 1.12, 95% CI: 1.04–1.20; aHR: 1.08, 95% CI: 1.03–1.14) (Table 2).

**Table 2:** Risks (aHR) of CVD in patients with gingivitis and tooth loss.

CVD type*		Gingivitis		Tooth loss		Gingivitis and Tooth loss	
		No (n = 2,795,298)	Yes (n = 984,192)	No (n = 3,095,860)	Yes (n = 683,630)	Neither (n = 2,357,343)	Both (n = 245,675)
Angina pectoris (n = 10,224)							
	N of events (%)	7,099 (69.4)	3,125 (30.6)	7,397 (72.3)	2,827 (27.7)	5,447 (53.3)	1,175 (11.5)
	Person-year	29,001,509	10,204,405	32,111,867	7,094,048	24,456,718	2,549,257
	aHR† (95% CI)	ref	1.02 (0.97–1.06)	ref	0.99 (0.95–1.04)	ref	1.04 (0.97–1.12)
Acute myocardial infarction (n = 6,182)							
	N of events (%)	4,260 (68.9)	1,922 (31.1)	4,466 (72.2)	1,716 (27.8)	3,259 (52.7)	715 (11.6)
	Person-year	29,014,010	10,209,501	32,124,462	7,099,049	24,466,170	2,551,209
	aHR† (95% CI)	ref	1.04 (0.98–1.10)	ref	1.01 (0.95–1.07)	ref	1.07 (0.98–1.17)
Stroke (n = 9,536)							
	N of events (%)	6,604 (69.3)	2,932 (30.7)	6,616 (69.4)	2,920 (30.6)	4,871 (51.1)	1,187 (12.5)
	Person-year	29,001,331	10,204,439	32,112,904	7,092,866	24,457,397	2,548,932
	aHR† (95% CI)	ref	1.03 (0.99–1.08)	ref	1.09 (1.04–1.15)	ref	1.12 (1.04–1.20)
Cardiovascular disease (n = 17,942)							
	N of events (%)	12,434 (69.3)	5,508 (30.7)	12,791 (71.3)	5,151 (28.7)	9,414 (52.5)	2,131 (11.9)
	Person-year	28,976,683	10,193,382	32,086,946	7,083,119	24,438,328	2,544,764
	aHR† (95% CI)	ref	1.03 (0.99–1.06)	ref	1.04 (1.00–1.07)	ref	1.08 (1.03–1.14)

*CVD; Cardiovascular disease

†aHR, adjusted hazard ratio; Adjusted for age, sex, income, BMI, alcohol intake, smoking, physical activity, hypertension, diabetes and total cholesterol

### 3. Age and sex stratified risks (aHR) of CVD in patients with gingivitis and tooth loss

The age and sex stratified analyses showed strong association between tooth loss, gingivitis, and G&T and CVD in participants aged over 50 years than in participants aged 20–49 years. In the group aged over 50 years, tooth loss was associated with higher risks for AP (aHR: 1.23, 95% CI: 1.17–1.28), AMI (aHR: 1.21, 95% CI: 1.14–1.28), ST (aHR: 1.38, 95% CI: 1.32–1.45), and CVD (aHR: 1.28, 95% CI: 1.24–1.33). Gingivitis was associated with higher risks for AP (aHR: 1.05, 95% CI: 1.01–1.10), ST (aHR: 1.05, 95% CI: 1.01–1.10), and CVD (aHR: 1.05, 95% CI: 1.02–1.09). G&T was associated with higher risks for AP (aHR: 1.31, 95% CI: 1.23–1.41), AMI (aHR: 1.30, 95% CI: 1.19–1.42), ST (aHR: 1.44, 95% CI: 1.35–1.54), and CVD (aHR: 1.36, 95% CI: 1.30–1.43). On the other hand, participants with both G&T, even if they were under 50 years of age, had a significantly higher risk of ST (aHR: 1.71, 95% CI: 1.13–2.58) and CVD (aHR: 1.39, 95% CI: 1.02–1.90), indicating the need for caution.

The association between tooth loss, gingivitis, and G&T and CVD was stronger in male participants than female participants. In the male group, tooth loss was associated with higher risk for ST (aHR: 1.12, 95% CI: 1.05–1.19) and G&T was associated with higher risks for ST (aHR: 1.15, 95% CI: 1.06–1.26 and CVD (aHR: 1.10, 95% CI: 1.03–1.17) (Table 3).

**Table 3:** Age and sex stratified risks (aHR) of CVD in patients with gingivitis and tooth loss.

CVD type*	Periodontal Disease		Age specific						Sex specific					
			Less than 50			50 or more			Male			Female		
			Non- cases/ CVD cases	aHR†	95% CI	Non- cases/ CVD cases	aHR†	95% CI	Non- cases/ CVD cases	aHR‡	95% CI	Non- cases/ CVD cases	aHR‡	95% CI
Angina pectoris (n = 10,224)	Gingivitis	No	1,012,777/451	ref		1,775,422/6,648	ref		1,693,522/5,430	ref		1,094,677/1,669	ref	
		Yes	272,239/157	1.05	0.86–1.28	708,828/2,968	1.05	1.01–1.10	618,299/2,415	1.02	0.97–1.08	362,768/710	1.01	0.91–1.11
	Tooth loss	No	1,160,078/547	ref		1,928,385/6,850	ref		1,883,175/5,664	ref		1,205,288/1,733	ref	
		Yes	124,938/61	0.89	0.67–1.19	555,865/2,766	1.23	1.17–1.28	428,646/2,181	1.01	0.95–1.06	252,157/646	0.95	0.86–1.05
	Gingivitis and Tooth loss	Neither	924,670/417	ref		1,427,226/5,030	ref		1,420,525/4,156	ref		931,371/1,291	ref	
		Both	36,831/27	1.08	0.68–1.71	207,669/1,148	1.31	1.23–1.41	155,649/907	1.06	0.98–1.15	88,851/268	1.00	0.86–1.15
Acute myocardial infarction (n = 6,182)	Gingivitis	No	1,012,927/301	ref		1,778,111/3,959	ref		1,695,438/3,514	ref		1,095,600/746	ref	
		Yes	272,278/118	1.18	0.93–1.49	709,992/1,804	1.06	1.00–1.12	619,102/1,612	1.05	0.98–1.12	363,168/310	0.99	0.86–1.15
	Tooth loss	No	1,160,249/376	ref		1,931,145/4,090	ref		1,885,132/3,707	ref		1,206,262/759	ref	
		Yes	124,956/43	0.91	0.64–1.28	556,958/1,673	1.21	1.14–1.28	429,408/1,419	1.01	0.94–1.08	252,506/297	0.98	0.85–1.14
	Gingivitis and Tooth loss	Neither	924,809/278	ref		1,429,275/2,981	ref		1,421,992/2,689	ref		932,092/570	ref	
		Both	36,838/20	1.14	0.66–1.96	208,122/695	1.30	1.19–1.42	155,962/594	1.08	0.98–1.19	88,998/121	1.02	0.82–1.26
Stroke (n = 9,536)	Gingivitis	No	1,012,832/396	ref		1,775,862/6,208	ref		1,694,861/4,091	ref		1,093,833/2,513	ref	
		Yes	272,250/146	1.23	1.00–1.51	709,010/2,786	1.05	1.01–1.10	618,827/1,887	1.05	0.99–1.11	362,433/1,045	1.01	0.96–1.09
	Tooth loss	No	1,160,153/472	ref		1,929,091/6,144	ref		1,884,774/4,065	ref		1,204,470/2,551	ref	
		Yes	124,929/70	1.21	0.92–1.59	555,781/2,850	1.38	1.32–1.45	428,914/1,913	1.12	1.05–1.19	251,796/1,007	1.05	0.97–1.14
	Gingivitis and Tooth loss	Neither	924,732/355	ref		1,427,740/4,516	ref		1,421,708/2,973	ref		930,764/1,898	ref	
		Both	36,829/29	1.71	1.13–2.58	207,659/1,158	1.44	1.35–1.54	155,761/795	1.15	1.06–1.26	88,727/392	1.06	0.94–1.19
Cardiovascular disease (n = 17,942)	Gingivitis	No	1,012,425/803	ref		1,770,439/11,631	ref		1,690,211/8,741	ref		1,092,653/3,693	ref	
		Yes	272,100/296	1.17	1.02–1.36	706,584/5,212	1.05	1.02–1.09	616,762/3,952	1.03	0.99–1.08	361,922/1,556	1.01	0.95–1.08
	Tooth loss	No	1,159,652/973	ref		1,923,417/11,818	ref		1,879,850/8,989	ref		1,203,219/3,802	ref	
		Yes	124,873/126	1.04	0.85–1.28	553,606/5,025	1.28	1.24–1.33	427,123/3,704	1.05	1.00–1.09	251,356/1,447	1.01	0.94–1.08
	Gingivitis and Tooth loss	Neither	924,355/732	ref		1,423,574/8,682	ref		1,418,098/6,583	ref		929,831/2,831	ref	
		Both	36,803/55	1.39	1.02–1.90	206,741/2,076	1.36	1.30–1.43	155,010/1,546	1.10	1.03–1.17	88,534/585	1.04	0.95–1.15

*CVD; Cardiovascular disease

aHR, adjusted hazard ratio; Adjusted for

†sex, income, BMI, alcohol intake, smoking, physical activity, hypertension, diabetes and total cholesterol

‡age, income, BMI, alcohol intake, smoking, physical activity, hypertension, diabetes and total cholesterol

### 4. Subgroup analyses of risks (aHR) of CVD in patients with gingivitis and tooth loss

The subgroup analyses for comorbidity and smoking status are presented in Tables 4 and 5. In the case of obese participants, all CVD risks tended to be higher compared to normal participants. In particular, among obese participants, those with tooth loss had a higher risk of ST (aHR: 1.12, 95% CI: 1.03–1.21), and those with G&T had a higher risk of CVD (aHR: 1.09, 95% CI: 1.01–1.19).

**Table 4:** Risks (aHR) of CVD in patients with gingivitis and tooth loss by comorbidities.

CVD type*	Periodontal Disease		Obesity						dyslipidemia					
			Normal			Obese			Normal			dyslipidemia		
			Non- cases/ CVD cases	aHR†	95% CI	Non- cases/ CVD cases	aHR†	95% CI	Non- cases/ CVD cases	aHR‡	95% CI	Non- cases/ CVD cases	aHR‡	95% CI
Angina pectoris (n = 10,224)	Gingivitis	No	1,926,461/3,965	ref		833,247/3,064	ref		2,492,635/5,712	ref		267,248/1,319	ref	
		Yes	651,400/1,726	1.02	0.96–1.08	321,651/1,378	1.02	0.95–1.09	867,806/2,470	1.00	0.95–1.05	106,290/638	1.10	0.98–1.22
	Tooth loss	No	2,134,653/4,139	ref		921,816/3,183	ref		2,762,104/5,873	ref		295,451/1,452	ref	
		Yes	443,208/1,552	0.97	0.91–1.03	233,082/1,259	1.03	0.96–1.11	598,337/2,309	1.01	0.96–1.06	78,087/505	0.93	0.82–1.04
	Gingivitis and Tooth loss	Neither	1,640,564/3,059	ref		685,787/2,331	ref		2,107,713/4,360	ref		218,823/1,030	ref	
		Both	157,311/646	1.01	0.92–1.11	85,622/526	1.09	0.98–1.21	213,415/957	1.05	0.97–1.13	29,662/216	1.00	0.84–1.19
Acute myocardial infarction (n = 6,182)	Gingivitis	No	1,928,084/2,342	ref		834,435/1,876	ref		2,495,007/3,340	ref		267,688/879	ref	
		Yes	652,100/1,026	1.02	0.94–1.10	322,148/881	1.06	0.97–1.16	868,802/1,474	1.01	0.95–1.08	106,494/434	1.16	1.01–1.32
	Tooth loss	No	2,136,369/2,423	ref		923,001/1,998	ref		2,764,539/3,438	ref		295,919/984	ref	
		Yes	443,815/945	0.98	0.90–1.07	233,582/759	1.04	0.95–1.14	599,270/1,376	1.02	0.95–1.09	78,263/329	0.94	0.81–1.10
	Gingivitis and Tooth loss	Neither	1,641,834/1,789	ref		686,680/1,438	ref		2,109,541/2,532	ref		219,158/695	ref	
		Both	157,565/392	1.04	0.92–1.17	85,827/321	1.12	0.97–1.28	213,804/568	1.06	0.96–1.17	29,733/145	1.13	0.91–1.39
Stroke (n = 9,536)	Gingivitis	No	1,926,408/4,018	ref		833,799/2,512	ref		2,492,726/5,621	ref		267,656/911	ref	
		Yes	651,355/1,771	1.05	1.00–1.12	321,890/1,139	1.01	0.93–1.09	867,767/2,509	1.04	0.98–1.09	106,524/404	1.02	0.89–1.17
	Tooth loss	No	2,134,759/4,033	ref		922,483/2,516	ref		2,762,344/5,633	ref		295,984/919	ref	
		Yes	443,004/1,756	1.08	1.01–1.15	233,206/1,135	1.12	1.03–1.21	598,149/2,497	1.09	1.03–1.15	78,196/396	1.13	0.98–1.31
	Gingivitis and Tooth loss	Neither	1,640,635/2,988	ref		686,290/1,828	ref		2,107,938/4,135	ref		219,171/682	ref	
		Both	157,231/726	1.13	1.03–1.24	85,697/451	1.10	0.98–1.23	213,361/1,011	1.11	1.03–1.20	29,711/167	1.15	0.93–1.42
Cardiovascular disease (n = 17,942)	Gingivitis	No	1,923,173/7,253	ref		831,262/5,049	ref		2,488,086/10,261	ref		266,524/2,043	ref	
		Yes	649,956/3,170	1.03	0.99–1.08	320,732/2,297	1.02	0.97–1.08	865,764/4,512	1.02	0.98–1.06	105,967/961	1.08	0.99–1.18
	Tooth loss	No	2,131,339/7,453	ref		919,792/5,207	ref		2,757,499/10,478	ref		294,717/2,186	ref	
		Yes	441,790/2,970	1.02	0.97–1.07	232,202/2,139	1.06	1.00–1.12	596,351/4,295	1.04	1.00–1.08	77,774/818	1.01	0.92–1.11
	Gingivitis and Tooth loss	Neither	1,638,102/5,521	ref		684,328/3,790	ref		2,104,337/7,736	ref		218,278/1,575	ref	
		Both	156,719/1,238	1.07	1.00–1.15	85,268/880	1.09	1.01–1.19	212,602/1,770	1.08	1.02–1.14	29,528/350	1.08	0.93–1.24

*CVD; Cardiovascular disease

aHR, adjusted hazard ratio; Adjusted for

†age, sex, income, alcohol intake, smoking, physical activity, hypertension, diabetes and total cholesterol

‡age, sex, income, BMI, alcohol intake, smoking, physical activity, hypertension, and diabetes

**Table 5:** Risks (aHR) of CVD in patients with gingivitis and tooth loss by smoking.

CVD type*	Periodontal Disease		Smoking					
			Never smoker			Smoker		
			Non-cases/ CVD cases	aHR †	95% CI	Non-cases/ CVD cases	aHR †	95% CI
Angina pectoris (n = 10,224)								
	Gingivitis	No	1,508,327/2,896	ref		1,229,824/4,081	ref	
		Yes	498,661/1,196	0.98	0.92–1.06	470,069/1,900	1.04	0.98–1.10
	Tooth loss	No	1,670,001/3,010	ref		1,364,497/4,264	ref	
		Yes	336,987/1,082	0.95	0.88–1.03	335,396/1,717	1.02	0.96–1.09
	Gingivitis and Tooth loss	Neither	1,289,926/2,243	ref		1,017,514/3,101	ref	
		Both	118,586/429	0.97	0.86–1.08	123,086/737	1.09	1.00–1.19
Acute myocardial infarction (n = 6,182)								
	Gingivitis	No	1,509,736/1,487	ref		1,231,210/2,695	ref	
		Yes	499,252/605	0.97	0.87–1.07	470,678/1,291	1.07	1.00–1.15
	Tooth loss	No	1,671,467/1,544	ref		1,365,918/2,843	ref	
		Yes	337,521/548	0.92	0.82–1.02	335,970/1,143	1.05	0.98–1.14
	Gingivitis and Tooth loss	Neither	1,291,011/1,158	ref		1,018,576/2,039	ref	
		Both	118,796/219	0.94	0.80–1.10	123,336/487	1.14	1.02–1.27
Stroke (n = 9,536)								
	Gingivitis	No	1,507,696/3,527	ref		1,230,956/2,949	ref	
		Yes	498,387/1,470	1.00	0.95–1.08	470,545/1,424	1.06	0.99–1.13
	Tooth loss	No	1,669,431/3,580	ref		1,365,844/2,917	ref	
		Yes	336,652/1,417	1.04	0.97–1.11	335,657/1,456	1.16	1.08–1.24
	Gingivitis and Tooth loss	Neither	1,289,505/2,664	ref		1,018,504/2,111	ref	
		Both	118,461/554	1.04	0.94–1.15	123,205/618	1.21	1.09–1.34
Cardiovascular disease (n = 17,942)								
	Gingivitis	No	1,505,500/5,723	ref		1,227,430/6,475	ref	
		Yes	497,475/2,382	1.00	0.95–1.06	468,909/3,060	1.05	1.00–1.10
	Tooth loss	No	1,667,101/5,910	ref		1,362,109/6,652	ref	
		Yes	335,874/2,195	0.99	0.94–1.04	334,230/2,883	1.07	1.02–1.13
	Gingivitis and Tooth loss	Neither	1,287,768/4,401	ref		1,015,789/4,826	ref	
		Both	118,142/873	1.01	0.93–1.09	122,589/1,234	1.14	1.07–1.22

*CVD; Cardiovascular disease

†aHR, adjusted hazard ratio; Adjusted for age, sex, income, BMI, alcohol intake, physical activity, hypertension, diabetes and total cholesterol

As a result of stratification according to dyslipidemia, no clear trend was found, but the risk of AMI was 16% higher in the gingivitis group with dyslipidemia (aHR: 1.16, 95% CI: 1.01–1.32). Even if the cholesterol level was normal, the risk of ST was still significantly high when they had tooth loss or G&T (aHR: 1.09, 95% CI: 1.03–1.15; aHR: 1.11, 95% CI: 1.03–1.20), and the risk of CVD was significantly high when they had G&T (aHR: 1.08, 95% CI: 1.02–1.14).

Among smokers, the risk of ST and CVD in the tooth loss group was high (aHR: 1.16, 95% CI: 1.08–1.24; aHR: 1.07, 95% CI: 1.02–1.13). In addition, the risk of AMI, ST, and CVD was high in the group with G&T (aHR: 1.14, 95% CI: 1.02–1.27; aHR: 1.21, 95% CI: 1.09–1.34; aHR: 1.14, 95% CI: 1.07–1.22). On the other hand, never-smokers did not have higher risks for any CVD events significantly.

## Discussion

In this large retrospective cohort study of Korea, a clear positive association between PD and the risk of ST and CVD was documented. Particularly, these associations were stronger in participants with male, those who were older (≥50 than <50), obesity, and smokers. After stratified by dyslipidemia, there was no consistent trend.

Most previous studies reported that PD increases the risk of CVD, and studies reported the magnitude of the risk to be between 1.10 and 1.30, which was similar to the 1.08 times in our study [6, 8, 25, 31, 32]. In some cases, studies showed values more than 2.0 times higher, but they appear to have been overestimated because many confounding factors were not controlled [33]. In particular, among the detailed diseases, there were many reports of ST. PD is thought to clearly increase the risk of ST, and the magnitude of the risk was similar, ranging from 1.12 in this study to 1.10–1.30 in other studies [8, 11, 17, 25, 32–35]. There was also a study that showed a high impact with a risk of more than 3 times, and this appears to be because the long observation period of more than 30 years and severe PD such as periodontal bone loss were independent variables [36].

Among the specific diseases, there are inconsistent results regarding the association between PD and AMI, and there is a lack of evidence for the association. Howell et al. [13] showed a weak association of 1.12, Noguchi et al. [37] showed a high risk of 2.26, and Lee et al. [38] showed a low risk of 0.95. All these studies were not statistically significant. On the other hand, Yu et al. [39] reported a high risk of 1.72 significantly, but PD was self-measured, and the study was conducted on a single gender (female) without adjusting for alcohol drinking, etc. Moreover, Rydén at al. [40] reported a high risk of 1.28. However, as the sample size was insufficient as a case-control study, it seems that well-designed large-scale data studies should be accumulated. In this study, most confounding factors were controlled to confirm the causal relationship between PD and specific CVD, and as a result of 10-year follow-up, a non-significant risk of 1.01–1.07 was observed, confirming that the association with PD and AMI was weak. Regarding AP, there are few studies on the association, and similar to this study, the results were not statistically significant [38].

The impact of PD on CVD, especially ST, presented in this study is likely to result from chronic inflammation caused by gingivitis and periodontitis. Chronic PD causes immune dysregulation and systemic inflammation, and the role of oral pathogens such as *P*. *gingivalis* has been suggested [21, 41–43]. Specifically, pathogenic microorganisms in periodontitis release inflammatory cytokines, chemokines, proteolytic enzymes, and reactive oxygen species through interactions with tissues and cells, causing local and irreversible degeneration of periodontal structures. Infectious agents and inflammatory mediators can spread throughout the body, causing inflammatory conditions and promoting the development of systemic diseases. In addition, interleukin (IL) 1β, IL-6, or tumor necrosis factor (TNF) are reported to affect cardiovascular disease by circulating throughout the body and causing responses in other tissues [43]. Additionally, it has recently been suggested that the atherosclerotic process that causes ST is an immune-metabolic response of blood vessels to various harmful factors [44, 45].

Meanwhile, as a result of sub-group analysis to control residual confounding effects, the effect was evident in older, male [46, 47], and smoking in particular appears to be a major factor increasing the effect [6, 10, 11, 25, 48]. PD and CVD are closely related as they share risk factors such as smoking, diabetes, obesity, lack of physical activity, and stress, so additional research is needed to uncover this connection [6, 9, 11]. In addition to age, an additional point to note is that although the effect is mostly large and shows a significant trend in the elderly, a few studies have shown a high risk of ST even in young participants [35]. In this study, only in the case of ST, the risk in participants under 50 years of age was 1.71 and significant. Managing PD will be very important to prevent ST in young people.

While numerous studies have explored the link between PD and CVD, analyses of specific CVD with each sub groups on all age adults are rare. One of the strengths of our study was that the large-scale data consisted of nearly 3.7 million participants who have undergone mandatory National oral examination for one year, so it is representative with no recall bias and little selection bias. This big data defines PD through clinical examinations by dentists and clinically defines CVD using health insurance claim data, so it has higher clinical reliability than self-measurement and surveys. In addition, we attempted to derive accurate results by correcting ten confounding variables, including comorbidities. Despite these strengths, this study has several limitations. Variables for PD were recorded in a binary format, so differences in disease severity could not be analyzed. Additionally, as tooth loss is a time-dependent variable, the cumulative effect due to chewing discomfort and deterioration in quality of life will change. Therefore, it is suggested that follow-up studies examine the effect of tooth loss according to the duration. Moreover, since the explanation of the mechanism for the association between the two diseases identified in this study could not be measured, further experimental study on mechanism is needed. Lastly, this study did not reflect the severity of CVD.

## Conclusions

Our results highlight an association between tooth loss, gingivitis, and the occurrence of CVD, specifically ST, emphasizing the critical need for preventive oral healthcare interventions. Additionally, our findings indicate the necessity for tailored interventions aimed at reducing the heightened risk of CVD events, particularly ST, among older, obese individuals and smokers.

## References

[1] Tu C, Wang G, Hu Z, Wang S, Yan Q, Liu X. Burden of oral disorders, 1990–2019: estimates from the Global Burden of Disease Study 2019. Arch Med Sci. 2023 Jul 13;19(4):930–940. doi: 10.5114/aoms/165962 ; PMCID: PMC10408023. 37560733 PMC10408023

[2] Kwon T, Lamster IB, Levin L. Current Concepts in the Management of Periodontitis. Int Dent J. 2021 Dec;71(6):462–476. doi: 10.1111/idj.12630 Epub 2021 Feb 19. ; PMCID: PMC9275292. 34839889 PMC9275292

[3] 2021. Most Frequent Disease Statistics. Health Insurance Review and Assessment Service, 2022. [Available at: https://opendata.hira.or.kr/op/opc/olapHifrqSickInfoTab1.do. Accessed October 15, 2023.]

[4] Haffajee AD, Socransky SS. Microbial etiological agents of destructive periodontal diseases. Periodontol 2000. 1994 Jun;5:78–111. doi: 10.1111/j.1600-0757.1994.tb00020.x . 9673164

[5] Scannapieco FA, Gershovich E. The prevention of periodontal disease-An overview. Periodontol 2000. 2020 Oct;84(1):9–13. doi: 10.1111/prd.12330 . 32844421

[6] LaMonte MJ, Genco RJ, Hovey KM, Wallace RB, Freudenheim JL, Michaud DS, et al. History of Periodontitis Diagnosis and Edentulism as Predictors of Cardiovascular Disease, Stroke, and Mortality in Postmenopausal Women. J Am Heart Assoc. 2017 Mar 29;6(4):e004518. doi: 10.1161/JAHA.116.004518 ; PMCID: PMC5532989. 28356279 PMC5532989

[7] de Oliveira C, Watt R, Hamer M. Toothbrushing, inflammation, and risk of cardiovascular disease: results from Scottish Health Survey. BMJ. 2010 May 27;340:c2451. doi: 10.1136/bmj.c2451 ; PMCID: PMC2877809. 20508025 PMC2877809

[8] Beck J, Garcia R, Heiss G, Vokonas PS, Offenbacher S. Periodontal disease and cardiovascular disease. J Periodontol. 1996 Oct;67(10 Suppl):1123–37. doi: 10.1902/jop.1996.67.10s.1123 8910831

[9] Senba T, Kobayashi Y, Inoue K, Kaneto C, Inoue M, Toyokawa S, et al. The association between self-reported periodontitis and coronary heart disease—from MY Health Up Study—. J Occup Health. 2008;50(3):283–7. doi: 10.1539/joh.l7066 Epub 2008 Apr 15. . 18413975

[10] Batty GD, Jung KJ, Mok Y, Lee SJ, Back JH, Lee S, et al. Oral health and later coronary heart disease: Cohort study of one million people. Eur J Prev Cardiol. 2018 Apr;25(6):598–605. doi: 10.1177/2047487318759112 Epub 2018 Feb 20. ; PMCID: PMC5946673. 29461088 PMC5946673

[11] Choe H, Kim YH, Park JW, Kim SY, Lee SY, Jee SH. Tooth loss, hypertension and risk for stroke in a Korean population. Atherosclerosis. 2009 Apr;203(2):550–6. doi: 10.1016/j.atherosclerosis.2008.07.017 2008.07.017. Epub 2008 Jul 26. . 19013571

[12] Beukers NGFM, van der Heijden GJMG, Su N, van der Galiën O, Gerdes VEA, Loos BG. An examination of the risk of periodontitis for nonfatal cardiovascular diseases on the basis of a large insurance claims database. Community Dent Oral Epidemiol. 2023 Jun;51(3):408–417. doi: 10.1111/cdoe.12752 Epub 2022 May 13. . 35561035

[13] Howell TH, Ridker PM, Ajani UA, Hennekens CH, Christen WG. Periodontal disease and risk of subsequent cardiovascular disease in U.S. male physicians. J Am Coll Cardiol. 2001 Feb;37(2):445–50. doi: 10.1016/s0735-1097(00)01130-x . 11216961

[14] Hansen GM, Egeberg A, Holmstrup P, Hansen PR. Relation of Periodontitis to Risk of Cardiovascular and All-Cause Mortality (from a Danish Nationwide Cohort Study). Am J Cardiol. 2016 Aug 15;118(4):489–93. doi: 10.1016/j.amjcard.2016.05.036 Epub 2016 May 30. . 27372888

[15] Ahn YB, Shin MS, Byun JS, Kim HD. The association of hypertension with periodontitis is highlighted in female adults: results from the Fourth Korea National Health and Nutrition Examination Survey. J Clin Periodontol. 2015 Nov;42(11):998–1005. doi: 10.1111/jcpe.12471 Epub 2015 Nov 14. . 26461204

[16] Lin HW, Chen CM, Yeh YC, Chen YY, Guo RY, Lin YP, et al. Dental treatment procedures for periodontal disease and the subsequent risk of ischaemic stroke: A retrospective population-based cohort study. J Clin Periodontol. 2019 Jun;46(6):642–649. doi: 10.1111/jcpe.13113 Epub 2019 May 7. . 30989681

[17] Sen S, Giamberardino LD, Moss K, Morelli T, Rosamond WD, Gottesman RF, et al. Periodontal Disease, Regular Dental Care Use, and Incident Ischemic Stroke. Stroke. 2018 Feb;49(2):355–362. doi: 10.1161/STROKEAHA.117.018990 Epub 2018 Jan 15. ; PMCID: PMC5780242. 29335336 PMC5780242

[18] Desvarieux M, Demmer RT, Jacobs DR, Papapanou PN, Sacco RL, Rundek T. Changes in clinical and microbiological periodontal profiles relate to progression of carotid intima-media thickness: the Oral Infections and Vascular Disease Epidemiology study. J Am Heart Assoc. 2013 Oct 28;2(6):e000254. doi: 10.1161/JAHA.113.000254 ; PMCID: PMC3886779. 24166489 PMC3886779

[19] Lockhart PB, Bolger AF, Papapanou PN, Osinbowale O, Trevisan M, Levison ME, et al. Periodontal disease and atherosclerotic vascular disease: does the evidence support an independent association?: a scientific statement from the American Heart Association. Circulation. 2012 May 22;125(20):2520–44. doi: 10.1161/CIR.0b013e31825719f3 Epub 2012 Apr 18. . 22514251

[20] Hajishengallis G. Periodontitis: from microbial immune subversion to systemic inflammation. Nat Rev Immunol. 2015 Jan;15(1):30–44. doi: 10.1038/nri3785 ; PMCID: PMC4276050. 25534621 PMC4276050

[21] Hayashi C, Gudino CV, Gibson FC 3rd, Genco CA. Review: Pathogen-induced inflammation at sites distant from oral infection: bacterial persistence and induction of cell-specific innate immune inflammatory pathways. Mol Oral Microbiol. 2010 Oct;25(5):305–16. doi: 10.1111/j.2041-1014.2010.00582.x ; PMCID: PMC2951292. 20883220 PMC2951292

[22] Geismar K, Stoltze K, Sigurd B, Gyntelberg F, Holmstrup P. Periodontal disease and coronary heart disease. J Periodontol. 2006 Sep;77(9):1547–54. doi: 10.1902/jop.2006.050405 . 16945033

[23] Roth GA, Mensah GA, Johnson CO, Addolorato G, Ammirati E, Baddour LM, et al.; GBD-NHLBI-JACC Global Burden of Cardiovascular Diseases Writing Group. Global Burden of Cardiovascular Diseases and Risk Factors, 1990–2019: Update From the GBD 2019 Study. J Am Coll Cardiol. 2020 Dec 22;76(25):2982–3021. doi: 10.1016/j.jacc.2020.11.010 Erratum in: J Am Coll Cardiol. 2021 Apr 20;77(15):1958–1959. ; PMCID: PMC7755038. 33309175 PMC7755038

[24] Okwuosa IS, Lewsey SC, Adesiyun T, Blumenthal RS, Yancy CW. Worldwide disparities in cardiovascular disease: Challenges and solutions. Int J Cardiol. 2016 Jan 1;202:433–40. doi: 10.1016/j.ijcard.2015.08.172 Epub 2015 Aug 28. . 26433167

[25] Abnet CC, Qiao YL, Dawsey SM, Dong ZW, Taylor PR, Mark SD. Tooth loss is associated with increased risk of total death and death from upper gastrointestinal cancer, heart disease, and stroke in a Chinese population-based cohort. Int J Epidemiol. 2005 Apr;34(2):467–74. doi: 10.1093/ije/dyh375 Epub 2005 Jan 19. . 15659476

[26] National Health Screening Act, Act No. 14462, Dec. 30, 2016. Available at: https://law.go.kr/LSW/lsInfoP.do?lsId=010708&ancYnChk=0#0000

[27] Kim BY, Kang SM, Kang JH, Kang SY, Kim KK, Kim KB, et al.; Committee of Clinical Practice Guidelines, Korean Society for the Study of Obesity (KSSO). 2020 Korean Society for the Study of Obesity Guidelines for the Management of Obesity in Korea. J Obes Metab Syndr. 2021 Jun 30;30(2):81–92. doi: 10.7570/jomes21022 ; PMCID: PMC8277596. 34045368 PMC8277596

[28] Kim HC, Ihm SH, Kim GH, Kim JH, Kim KI, Lee HY, et al. 2018 Korean Society of Hypertension guidelines for the management of hypertension: part I-epidemiology of hypertension. Clin Hypertens. 2019 Aug 1;25:16. doi: 10.1186/s40885-019-0121-0 ; PMCID: PMC6670210. 31388451 PMC6670210

[29] Jeon JY, Ko SH, Kwon HS, Kim NH, Kim JH, Kim CS, et al.; Taskforce Team of Diabetes Fact Sheet of the Korean Diabetes Association. Prevalence of Diabetes and Prediabetes according to Fasting Plasma Glucose and HbA1c. Diabetes Metab J. 2013 Oct;37(5):349–57. doi: 10.4093/dmj.2013.37.5.349 Epub 2013 Oct 17. ; PMCID: PMC3816136. 24199164 PMC3816136

[30] Rhee EJ, Kim HC, Kim JH, Lee EY, Kim BJ, Kim EM, et al.; Committee of Clinical Practice Guideline of Korean Society of Lipid and Atherosclerosis. 2018 Guidelines for the Management of Dyslipidemia in Korea. J Lipid Atheroscler. 2019 Sep;8(2):78–131. doi: 10.12997/jla.2019.8.2.78 Epub 2019 Aug 7. ; PMCID: PMC7379116. 32821702 PMC7379116

[31] Joshy G, Arora M, Korda RJ, Chalmers J, Banks E. Is poor oral health a risk marker for incident cardiovascular disease hospitalisation and all-cause mortality? Findings from 172 630 participants from the prospective 45 and Up Study. BMJ Open. 2016 Aug 30;6(8):e012386. doi: 10.1136/bmjopen-2016-012386 ; PMCID: PMC5013478. 27577588 PMC5013478

[32] Mucci LA, Hsieh CC, Williams PL, Arora M, Adami HO, de Faire U, et al. Do genetic factors explain the association between poor oral health and cardiovascular disease? A prospective study among Swedish twins. Am J Epidemiol. 2009 Sep 1;170(5):615–21. doi: 10.1093/aje/kwp177 Epub 2009 Jul 31. ; PMCID: PMC2732988. 19648170 PMC2732988

[33] Morrison HI, Ellison LF, Taylor GW. Periodontal disease and risk of fatal coronary heart and cerebrovascular diseases. J Cardiovasc Risk. 1999 Feb;6(1):7–11. doi: 10.1177/204748739900600102 . 10197286

[34] Joshipura KJ, Hung HC, Rimm EB, Willett WC, Ascherio A. Periodontal disease, tooth loss, and incidence of ischemic stroke. Stroke. 2003 Jan;34(1):47–52. doi: 10.1161/01.str.0000052974.79428.0c . 12511749

[35] Lee YL, Hu HY, Huang N, Hwang DK, Chou P, Chu D. Dental prophylaxis and periodontal treatment are protective factors to ischemic stroke. Stroke. 2013 Apr;44(4):1026–30. doi: 10.1161/STROKEAHA.111.000076 Epub 2013 Feb 19. . 23422085

[36] Jimenez M, Krall EA, Garcia RI, Vokonas PS, Dietrich T. Periodontitis and incidence of cerebrovascular disease in men. Ann Neurol. 2009 Oct;66(4):505–12. doi: 10.1002/ana.21742 ; PMCID: PMC2783821. 19847898 PMC2783821

[37] Noguchi S, Toyokawa S, Miyoshi Y, Suyama Y, Inoue K, Kobayashi Y. Five-year follow-up study of the association between periodontal disease and myocardial infarction among Japanese male workers: MY Health Up Study. J Public Health (Oxf). 2015 Dec;37(4):605–11. doi: 10.1093/pubmed/fdu076 Epub 2014 Oct 7. . 25293424

[38] Lee JH, Oh JY, Youk TM, Jeong SN, Kim YT, Choi SH. Association between periodontal disease and non-communicable diseases: A 12-year longitudinal health-examinee cohort study in South Korea. Medicine (Baltimore). 2017 Jun;96(26):e7398. doi: 10.1097/MD.0000000000007398 ; PMCID: PMC5500097. 28658175 PMC5500097

[39] Yu YH, Chasman DI, Buring JE, Rose L, Ridker PM. Cardiovascular risks associated with incident and prevalent periodontal disease. J Clin Periodontol. 2015 Jan;42(1):21–8. doi: 10.1111/jcpe.12335 Epub 2015 Jan 9. ; PMCID: PMC4300240. 25385537 PMC4300240

[40] Rydén L, Buhlin K, Ekstrand E, de Faire U, Gustafsson A, Holmer J, et al. Periodontitis Increases the Risk of a First Myocardial Infarction: A Report From the PAROKRANK Study. Circulation. 2016 Feb 9;133(6):576–83. doi: 10.1161/CIRCULATIONAHA.115.020324 Epub 2016 Jan 13. . 26762521

[41] Schmidt J, Jentsch H, Stingu CS, Sack U. General immune status and oral microbiology in patients with different forms of periodontitis and healthy control subjects. PLoS One. 2014 Oct 9;9(10):e109187. doi: 10.1371/journal.pone.0109187 ; PMCID: PMC4192146. 25299619 PMC4192146

[42] Cifcibasi E, Ciblak M, Kiran B, Badur S, Firatli E, Issever H, et al. The role of activated cytotoxic T cells in etiopathogenesis of periodontal disease: does it harm or does it heal? Sci Rep. 2015 Mar 19;5:9262. doi: 10.1038/srep09262 ; PMCID: PMC4365406. 25788457 PMC4365406

[43] Friedewald VE, Kornman KS, Beck JD, Genco R, Goldfine A, Libby P, et al. The American Journal of Cardiology and Journal of Periodontology Editors’ Consensus: periodontitis and atherosclerotic cardiovascular disease. Am J Cardiol. 2009 Jul 1;104(1):59–68. doi: 10.1016/j.amjcard.2009.05.002 . 19576322

[44] Cho HJ, Shin MS, Song Y, Park SK, Park SM, Kim HD. Severe Periodontal Disease Increases Acute Myocardial Infarction and Stroke: A 10-Year Retrospective Follow-up Study. J Dent Res. 2021 Jul;100(7):706–713. doi: 10.1177/0022034520986097 Epub 2021 Jan 21. . 33478309

[45] Gao T, Zhang Z, Yu W, Zhang Z, Wang Y. Atherosclerotic carotid vulnerable plaque and subsequent stroke: a high-resolution MRI study. Cerebrovasc Dis. 2009;27(4):345–52. doi: 10.1159/000202011 Epub 2009 Feb 14. ; PMCID: PMC2814027. 19218800 PMC2814027

[46] Desvarieux M, Schwahn C, Völzke H, Demmer RT, Lüdemann J, Kessler C, et al. Gender differences in the relationship between periodontal disease, tooth loss, and atherosclerosis. Stroke. 2004 Sep;35(9):2029–35. doi: 10.1161/01.STR.0000136767.71518.36 Epub 2004 Jul 15. . 15256677

[47] Grau AJ, Buggle F, Ziegler C, Schwarz W, Meuser J, Tasman AJ, et al. Association between acute cerebrovascular ischemia and chronic and recurrent infection. Stroke. 1997 Sep;28(9):1724–9. doi: 10.1161/01.str.28.9.1724 . 9303015

[48] Syrjälä AM, Ylöstalo P, Hartikainen S, Sulkava R, Knuuttila ML. Number of teeth and myocardial infarction and stroke among elderly never smokers. J Negat Results Biomed. 2009 Apr 22;8:6. doi: 10.1186/1477-5751-8-6 ; PMCID: PMC2675514. 19386093 PMC2675514

